# Edaravone for the Treatment of Motor Neurone Disease: A Critical Review of Approved and Alternative Formulations against a Proposed Quality Target Product Profile

**DOI:** 10.3390/pharmaceutics16080993

**Published:** 2024-07-26

**Authors:** Riuna O’Neill, Okhee Yoo, Philip Burcham, Lee Yong Lim

**Affiliations:** 1 Division of Pharmacy, School of Allied Health, University of Western Australia, Perth, WA 6009, Australia; riuna.oneill@research.uwa.edu.au (R.O.); okhee.yoo@uwa.edu.au (O.Y.); philip.burcham@uwa.edu.au (P.B.); 2 Institute for Paediatric Perioperative Excellence, University of Western Australia, Perth, WA 6009, Australia; 3 Wesfarmers Centre of Vaccines and Infectious Diseases, Telethon Kids Institute, University of Western Australia, Perth, WA 6009, Australia; 4 Division of Pharmacology and Toxicology, School of Biomedical Science, University of Western Australia, Perth, WA 6009, Australia

**Keywords:** edaravone, MND, ALS, formulation, preparation, excipients, solubility, pharmacokinetics, bioavailability

## Abstract

Edaravone is one of two main drugs for treating motor neurone disease (MND). This review proposes a specific quality target product profile (QTPP) for edaravone following an appraisal of the issues accounting for the poor clinical uptake of the approved IV and oral liquid edaravone formulations. This is followed by a review of the alternative oral formulations of edaravone described in the published patent and journal literature against the QTPP. A total of 14 texts published by six research groups on 18 novel oral formulations of edaravone for the treatment of MND have been reviewed. The alternative oral formulations included liquid and solid formulations developed with cyclodextrins, lipids, surfactants, co-surfactants, alkalising agents, tablet excipients, and co-solvents. Most were intended to deliver edaravone for drug absorption in the lower gastrointestinal tract (GIT); however, there were also four formulations targeting the oral mucosal absorption of edaravone to avoid first-pass metabolism. All the novel formulations improved the aqueous solubility, stability, and oral bioavailability (BA) of edaravone compared to an aqueous suspension of edaravone. A common limitation of the published formulations is the lack of MND-patient-centred data. Except for TW001, no other formulations have been trialled in MND patients. To meet the QTPP of an oral edaravone formulation for MND patients, it is recommended that a tablet of appropriate size and with acceptable taste and stability be designed for the effective sublingual or buccal absorption of edaravone. This tablet should be designed with input from the MND community.

## 1. Introduction

Motor neurone disease (MND) is a progressive disease, wherein the motor neurones in the brain and spinal cord undergo degeneration [1,2,3,4]. The neuroprotective drug edaravone (3-methyl-1-phenyl-2-pyrazolin-5-one, C10H10N2O) has strong antioxidant activities and can slow motor neurone degeneration and MND disease progression by 33% [4,5,6,7]. Currently, only the IV and oral suspension formulations of edaravone are commercially available. The clinical uptake of these formulations has been limited due to administration challenges [8,9]. Multiple attempts have since been made to design alternative peroral and oral mucosal formulations of edaravone; however, the novel formulations are yet to be reviewed against the approved edaravone products or the specific needs of MND patients.

**Figure 1:**
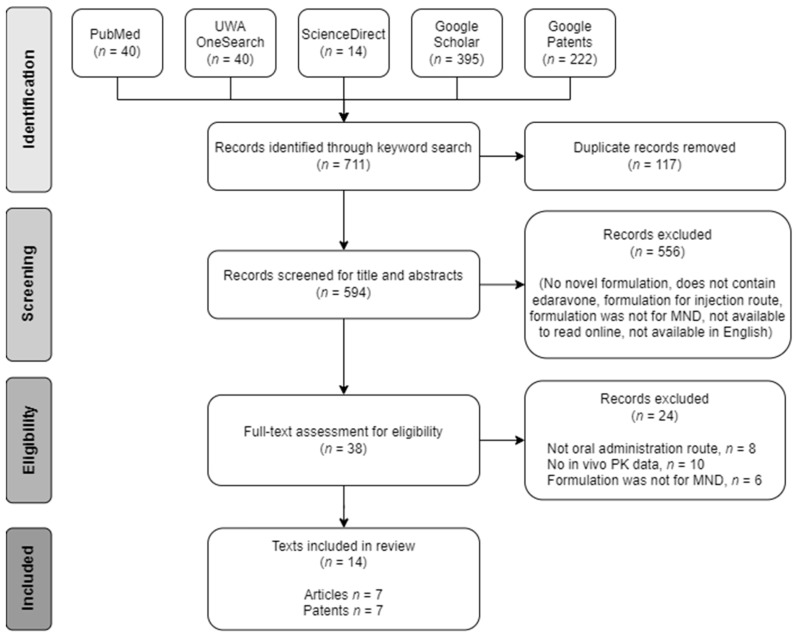
Flow diagram of search procedure for the published literature on alternative oral edaravone formulations extracted for this review.

The alternative edaravone formulations examined in this review were obtained from the published patent and journal literature. The inclusion criteria were publicly accessible records published in English on novel oral edaravone formulations for MND treatment that provide information on the formulation composition, preparation method, and pharmacokinetic (PK) data. A total of 711 records were identified through searches on PubMed, the University of Western Australia’s OneSearch database, ScienceDirect, Google Scholar, and Google Patents using the keyworks, “edaravone” and “formulation” or “preparation” (Figure 1). Of these, 117 duplicate records were removed, while a further 556 records were deemed inappropriate following examination of the title and abstract. Full-text screens were applied on the remaining 38 records, and a further 24 were excluded that did not meet the inclusion criteria. The final number of records included for data extraction was 14, and the oral edaravone formulations described within were examined for their effects on the aqueous solubility and first-pass metabolism of edaravone. These variables have been established to have a significant impact on the oral bioavailability (BA) of the edaravone. PK data were reviewed to enable comparisons between the alternative formulations and the approved Radicava^®^ and Radicava ORS^®^ products. Additionally, as MND patients eventually present with limited mobility and dysphagia, specific MND-patient-centred factors were also considered in the review of the formulations.

The aim of this review is firstly to consider the challenges that MND patients currently face with edaravone administration and to develop a quality target product profile (QTPP) for edaravone medicinal products aimed at treating MND patients. The second aim is to examine whether the alternative oral edaravone formulations in the published literature meet the characteristics listed in the QTPP. This review concludes with a proposed formulation for edaravone aimed at satisfying the criteria in the QTPP. 

## 2. Motor Neurone Disease

MND has been classified into four diseases based on the onset and pattern of motor neurone degeneration [1]. These are amyotrophic lateral sclerosis (ALS), progressive bulbar palsy (PBP), progressive muscular atrophy (PMA), and primary lateral sclerosis (PLS). ALS (also known as Lou Gehrig’s disease) is the most common and is used in the USA and Japan to describe MND [2], while PBP is described as bulbar onset ALS in these countries [4,10]. ALS shows considerable variability in age at onset, site of onset, and disease progression. It is a multisystem neurodegenerative disorder with clinical, genetic, and neuropathological heterogeneity [11]. About 50% of patients have extra-motor manifestations, 10–15% are diagnosed with frontotemporal dementia (FTD), and 35–40% exhibit mild behavioural or cognitive changes [12]. In ALS and PBP, both the upper and lower motor neurones are involved, whereas only the lower motor neurones are affected in PMA, and only the upper motor neurones are affected in PLS [1]. ALS is differentiated from PBP by its characteristic muscle weakness and wasting in the limbs at first clinical presentation, while PBP symptoms begin in the muscles for speech and swallowing. PMA patients experience slower rates of disease progression compared to ALS and PBP, and it may be misdiagnosed in ALS patients due to similar disease onsets. PLS is rare and characterised by slow and effortful movements in the legs, followed by the body, arms, and finally the face. Regardless of the pattern of motor neurone degeneration, MND patients at the initial stage of disease presentation often experience mild symptoms, such as cramps, weak or stiff muscles, difficulties in speaking or swallowing, or unstable emotional responses [1,2]. These symptoms progressively worsen within months, resulting in paralysis, respiratory failure, and finally death [2]. MND is always fatal; the average survival time is 2.5 years following diagnosis, although around 5–10% of patients may live for over 10 years [1,2].

The worldwide MND incidence rate is relatively low, at two per one hundred thousand, which translates to approximately one hundred and forty thousand new diagnoses per year [1]. There is currently no cure for MND. The underlying cause of MND is also not clear. Only 10% of MND cases are familial and have a potential link to genetic causes, whereas 90% are sporadic cases with no clear aetiology [1,4]. Factors that cause the degenerative motor neurone damage associated with MND are understood to include free radical damage [1,2], protein aggregation [13], glutamate toxicity [14], mitochondrial dysfunction [15], dysfunctional signalling pathways [16], immune-mediated damage [17], glial cell pathology, RNA metabolism, and the loss of growth factors to maintain motor neurone survival [18,19]. Some of these factors have become targets for developing therapeutic agents for MND. 

## 3. Therapeutic Agents for MND

The USA FDA has to date approved eight medicinal products for the treatment of ALS: Qalsody^®^, Relyvrio^®^, Nuedexta^®^, Rilutek^®^, Tiglutik^®^, Exservan^®^, Radicava^®^, and Radicava ORS^®^ (summarised in Table 1) [20]. Qalsody^®^ was granted accelerated approval in 2023, and it contains the antisense oligonucleotide tofersen that targets the SOD-1 mutation [21]. Qalsody^®^ is required at three initial doses at 14-day intervals followed by a maintenance dose every 28 days, with the doses having to be administered by lumbar puncture by experienced healthcare professionals [22]. Relyvrio^®^ is a powder formulation comprising sodium phenylbutyrate and taurursodiol to be swallowed with water or administered via a feeding tube [23]. Relyvrio^®^ was fast-tracked for approval in 2022 after only Phase 2 trials [24]; however, following the March 2024 announcement of disappointing topline results for a Phase 3 trial [25], Relyvrio^®^ has been voluntarily withdrawn from the market [20]. Nuedexta^®^ capsules contain a fixed-dose combination of dextromethorphan hydrobromide and quinidine sulfate and were approved in 2010 for the treatment of pseudobulbar affect symptoms associated with neurological disease conditions, including MND [26]. 

The two drugs that are the mainstay of treatment specifically for MND are riluzole (introduced in 1995) and edaravone (2017) [1,2]. Riluzole slows MND disease progression by inhibiting glutamate release from neurones [10], and it is the active ingredient in Rilutek^®^ (50 mg conventional tablet), Tiglutik^®^ (50 mg/10 mL suspension, thickened to ease swallowing), and Exservan^®^ (50 mg rapidly dissolving oral film). Riluzole is taken orally at 50 mg every 12 h on an empty stomach [27,28,29]. Systematic reviews of randomised clinical trials suggest that riluzole can prolong the median survival time of MND patients by 2 to 19 months [30,31]. However, riluzole is effective only in the late stage of ALS presentation, the drug having no or unknown effects on earlier disease stages [32]. In contrast, edaravone is efficacious in the early stages of MND and may prolong the median survival time by 24 months relative to placebo control [33]. More importantly, a pivotal 6-month, randomised, placebo-controlled, double-blind study found edaravone to decrease the rate of physical functional decline in ALS patients by approximately 33%, particularly when treatment was initiated in the early stages of the disease [5,7]. Combined therapy of riluzole with edaravone has been shown to be more effective than riluzole alone in slowing MND progression [34,35], and this combination is now recommended for MND treatment. As riluzole is already available in convenient oral dosage forms, this review focuses on edaravone, which has presented challenges for formulating into oral medicines appropriate for MND patients.

**Table 1:** Medicinal products approved by USA FDA for amyotrophic lateral sclerosis (ALS) treatment.

Product Name	Active Ingredient/s	Formulation Type	Administration Method	Dose	Dosing Regimen	Ref.
Qualsody^®^	Tofersen	Solution	Intrathecal injection	100 mg/15 mL	First 3 doses at 14-day intervals; subsequent doses at 28-day intervals.	[22]
Relyvrio^®^	Sodium phenylbutyrate and taurursodiol	Oral suspension (packaged as a powder)	Oral	3 g sodium phe-nylbutyrate and 1 g taurursodiol	Once daily for first three weeks, then twice daily thereafter. Taken before a snack or meal.	[23]
Nuedexta^®^	Dextromethorphan hydrobromide and quinidine sulfate	Capsule	Oral	Dextrome-thorphan 20 mg and quinidine 10 mg	Once daily for first 7 days, then once every 12 h thereafter.	[26]
Rilutek^®^	Riluzole	Tablet	Oral	50 mg	Twice daily, at least 1 h before or 2 h after a meal.	[28]
Tiglutik^®^	Riluzole	Oral suspension	Oral	50 mg/10 mL	Twice daily, at least 1 h before or 2 h after a meal.	[29]
Exservan^®^	Riluzole	Rapidly dissolving oral film	Oral	50 mL	Twice daily, at least 1 h before or 2 h after a meal.	[27]
Radicava^®^	Edaravone	Solution	IV injection	30 mg/100 mL	60 mg infused over 60 min. Initial treatment cycle of daily dosing for 14 days, followed by a 14-day drug-free period. Subsequent cycles require 10 days of daily dosing out of 14-day periods, followed by a 14-day drug-free period.	[5]
Radicava ORS^®^	Edaravone	Oral suspension	Oral	105 mg/5 mL	Initial treatment cycle of daily dosing for 14 days, followed by a 14-day drug-free period. Subsequent cycles require 10 days of daily dosing out of 14-day periods, followed by a 14-day drug-free period.	[5]

## 4. Edaravone

**Figure 2:**
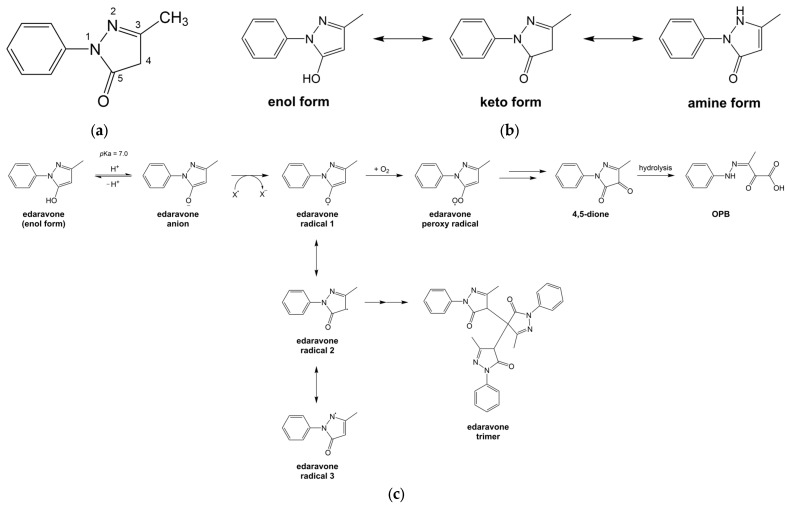
Edaravone: (**a**) chemical structure; (**b**) different forms of edaravone; and (**c**) mechanism of antioxidant activity. Edaravone is a bicyclic compound, consisting of a 2-pyrazolin-5-one ring with phenyl and methyl groups at positions 1 and 3, respectively. When ionised, the edaravone anion donates an electron to convert highly reactive ROS (X^•^) into more stable anions (X^−^). The newly formed edaravone radical may react with other edaravone radicals to form edaravone trimers [38] or is further oxidised to form a nontoxic stable compound, OPB [6].

Edaravone (Figure 2, 174.2 g/mol, melting point 129.7 °C) is an amphiphilic 2-pyrazolin-5-one derivative [36]. It is a crystalline powder that is freely soluble in ethanol but has limited solubility in water [5]. Edaravone is a weak acid (pK_a_ 6.9). At a low pH of 2 to 5, the nonionised edaravone has low solubility (~1.6 mg/mL). The solubility increases progressively with pH as edaravone becomes ionised, reaching 5.8 mg/mL at pH 10. Solid edaravone is in the keto form and is highly stable. In aqueous media, edaravone exists also in enol form and is especially unstable at pH 7 or higher when the edaravone anion predominates [8]. The edaravone anion can become a free radical by donating an electron to other free radicals, including molecular oxygen, causing the formation and precipitation of edaravone trimers (Figure 2). Edaravone in solution is stabilised by lowering the pH to inhibit ionisation and deoxygenation to reduce edaravone radical formation. Sodium bisulfite has been used as stabiliser, as it lowers solution pH and can also reduce edaravone self-association through adduct formation [37]. L-cysteine is another common stabiliser of edaravone formulations, even though it has not been found to stabilise edaravone solutions when oxygen is present [38]. 

Edaravone was synthesised in the late 1980s in Japan [4] and first approved for clinical use in 2001, for the treatment of acute ischaemic stroke [39,40]. It was approved for the treatment of MND patients in 2015, first in Japan and South Korea, then in the US (2017) [41], China (2019) [6,42], and Australia (2023) [43].

The mechanism of action of edaravone in MND, while uncertain, is generally agreed to be associated with its strong antioxidant properties [4]. Edaravone by electron donation can eliminate lipid peroxides and other reactive oxygen species (ROS), including hydroxyl (•OH) and peroxyl radicals (Figure 2). There is also evidence supporting its anti-inflammatory activities via the Nrf2/HO-1 and NFκB signalling pathways [6]. Additionally, edaravone forms Michael adducts with reactive carbonyl species to inhibit the carbonylation of functional and structural proteins [44]. The collective actions of edaravone, paired with its ability to cross the blood–brain barrier, are postulated to prevent further damage to the motor neurones in the central nervous system, thereby limiting disease progression and extending the lifespan of MND patients, especially when treatment is initiated soon after first diagnosis [7].

As of April 2024, the US, Canada, Japan, and Switzerland have approved two edaravone products for use in MND patients: an aqueous solution for IV administration (Radicava^®^) and a liquid suspension for oral administration (Radicava ORS^®^) [41,45]. In other countries, such as Australia and South Korea, only the IV formulation is available [43,46]. Both Radicava^®^ and Radicava ORS^®^ are registered by the Mitsubishi Tanabe Pharma Corporation, which also supplies the two products under other brand names, e.g., Radicut^®^ [47,48]. The treatment regimen is the same for both the IV and oral formulations, involving an initial treatment cycle of daily dosing for 14 days followed by 14 drug-free days and subsequent cycles requiring 10 days of daily dosing in a 14-day period followed by 14 drug-free days. 

Radicava^®^ is a 100 mL sterile isotonic aqueous solution in a single-dose polypropylene bag containing 30 mg of edaravone with 30 mg L-cysteine hydrochloride and 20 mg sodium bisulfite and adjusted to acidic pH 4 with phosphoric acid and sodium hydroxide. The polypropylene bag is overwrapped with polyvinyl alcohol secondary packaging that contains an oxygen absorber to minimise oxidation. Once the overwrap is opened, the product must be used within 24 h. Radicava^®^ is administered as an IV infusion, with two infusion bags administered over 60 min to provide the recommended edaravone dose of 60 mg.

Radicava ORS^®^ is a white aqueous suspension (pH 4) that contains 105 mg/5 mL of edaravone in a multi-dose amber glass bottle. The inactive ingredients are L-cysteine hydrochloride, polyvinyl alcohol, simethicone emulsion, sodium bisulfite, sorbitol, xanthan gum, phosphoric acid, and sodium hydroxide. The recommended dose is 5 mL, administered via oral syringe or feeding tube in the morning after overnight fasting. Radicava ORS^®^ can be stored at 20–25 °C; however, it must be used within 15 days after opening or within 30 days from the date of shipment from the manufacturer. It is supplied as two bottles each containing 35 mL for the first treatment cycle and as a single bottle containing 50 mL for subsequent treatment cycles [5].

## 5. Limitations of Approved Edaravone Dosage Forms

Prior to the introduction of Radicava ORS^®^, MND patients received edaravone only by IV infusion. Despite proven efficacy, the IV infusion has low clinical uptake [9]. Brooks et al. [49] found that only 2.5% (320 out of 12,892) of ALS patients in the USA had received edaravone between August 2017 and March 2020. Patients may have chosen not to receive Radicava^®^ because of the cost, trauma, and inconvenience of frequent IV infusions [50]. IV infusion requires administration by trained healthcare professionals, and the hospital/professional caregiver visits can incur significant physical and financial burdens on patients [51], with hospital visits becoming increasingly challenging when the patients’ ambulatory function worsens with disease progression [7]. Frequent IV infusions also have a high potential to cause device-related and injection-site infections [3]. 

Radicava ORS^®^ is taken orally which, compared to IV infusion, is more acceptable to patients because it is more convenient to administer, with lower product cost and lesser reliance on carers [8,52]. As dysphagia affects 85% of MND patients [4], Radicava ORS^®^ as a liquid formulation also facilitates swallowing [8]. However, for patients with severe dysphagia where the intake of 5 mL of liquid can risk severe coughing, aspiration into the lungs, and choking [53], Radicava ORS^®^ will require administration through a feeding tube. Radicava ORS^®^ is limited by its short shelf life of only one month, primarily due to the poor stability of edaravone in aqueous media especially when exposed to oxygen. The short shelf life requires the regular filling of prescriptions, with attendant costs associated with frequent shipping and packaging. Radicava ORS^®^ also has low oral BA of 57% relative to IV Radicava^®^ due to first-pass elimination by multiple uridine diphosphate glucuronosyltransferase (UGT) isoforms and sulfotransferase enzymes and incomplete dissolution of the edaravone particles in the suspension when delivered into the gastrointestinal tract (GIT) [52,54]. Additionally, patients have complained that Radicava ORS^®^ has unacceptable taste although the formulation contains sorbitol and xanthan gum, two common taste modifiers used in oral medicines. Another limitation, which applies to both Radicava ORS^®^ and Radicava^®^, is the presence of sodium bisulfite, a stabilising agent that can cause allergic reactions, including anaphylaxis and asthma, in hypersensitive patients [3,5].

## 6. Novel Oral Edaravone Formulations for MND Patients

### 6.1. Overview

Alternative oral edaravone formulations described in 14 published literature sources from six research groups were reviewed. These formulations were published between 2010 and 2024 and utilised several approaches in common to promote edaravone oral BA, mainly by addressing the solubility, dissolution, and stability of edaravone in aqueous media and its metabolism and efflux in the GIT. The approaches, summarised in Table 2, included complexation with water-soluble cyclodextrin derivatives, lipid-based self-microemulsifying drug delivery systems (SMEDDSs), solid dispersions, and the addition of alkalising, wetting, and/or solubilising agents. Some groups developed a series of similar formulations; however, only those formulations (Table 2) for which further physicochemical characterisations and oral BA had been determined are included in this review. It should be noted that this review includes the patent literature, which may not meet the stringent scientific standards of peer-reviewed pharmaceutical journals. 

The oral edaravone formulations consisted of liquids, powders, and tablets. Liquids were aqueous or lipid-based, the latter requiring containment in capsules for administration. Powder formulations were reconstituted into liquids for administration or recommended for further reformulation into tablets and capsules. Most formulations were intended to be swallowed with drug absorption occurring in the lower GIT [55,56]. Three groups provided formulations for oral mucosal delivery [57,58], including delivery under the tongue for drug absorption across the sublingual mucosa [59]. Most groups applied well-established pharmaceutical excipients to design and manufacture their formulations. The exception is the novel hydroxypropyl-sulfobutyl-β-cyclodextrin (HP-SBE-βCD) for forming water-soluble complexes with edaravone [60,61]. 

**Table 2:** Summary of alternative oral edaravone formulations.

Reference	Formulation Type	Description of Formulation
Peroral formulations for MND patients		
Ren et al., 2012 [60]; Zeng et al., 2011 [62]; Rong et al., 2014 [61]	Edaravone complexed with β-cyclodextrin (βCD)	Powder prepared by mixing solutions of edaravone in ethanol and βCD in phosphate buffer or water and freeze drying after ethanol evaporation. (βCD = βCD, hydroxypropyl (HP)-βCD, sulfobutyl (SBE)-βCD, HP-SBE-βCD).
Zhou et al., 2021 [56]; Parikh et al., 2016–2018 [8,52,63]	Liquid lipid-based self-microemulsifying drug delivery systems (SMEDDSs)	2. Liquid prepared by dissolving 10 mg/mL edaravone in a vehicle comprising by weight 30% Capryol™ PGMC (oil), 50% Cremophor^®^ RH 40–Labrasol^®^–D-α-tocopheryl polyethylene glycol 1000 succinate (TPGS 1000) (1:0.8:0.2, surfactants), and 20% Transcutol P^®^ (co-surfactant).
	Solid SMEDDS	3. Powder prepared by adding 1:1 Aerosil^®^ 200 to adsorb liquid SMEDDS in (2), followed by freeze drying.
	Solid dispersion	4. Powder prepared by solubilising edaravone 100 mg in ethanol with 500 mg of Soluplus^®^, evaporating off ethanol and crushing resultant film into powder.
	Aqueous solution with co-solvent/solubiliser	5. Liquid prepared by dissolving edaravone (3% *w/v*) in vehicle comprising an unspecified ratio of Labrasol^®^ and pH 4 citric acid buffer.
Moolenaar, 2019 [64]; Van Der Geest and Moolenaar, 2020 [55]; Moolenaar and Van Der Geest, 2021 [65]	Alkalinised solution	6. Liquid freshly prepared by dissolving 750 mg of powder formulation, comprising by weight 8.0% micronised edaravone, 58.2% mannitol, 33.3% sodium orthophosphate, and 0.5% sodium lauryl sulfate, in 50 mL water. 7. Liquid (pH 6.8–8.5) freshly prepared by dissolving in water 1.5% *w/v* of powder formulation, comprising by weight 9.3% micronised edaravone, 56.8% mannitol, 33.3% sodium orthophosphate, and 0.5% sodium lauryl sulfate.
Oral mucosal formulations for MND patients		
Sato et al., 2010 [57]	Aqueous solution with HP-βCD and antioxidants	8. Solution, 100 mL containing 1 g edaravone, 20 g HP-βCD, 1.2 g sodium bisulfite, and 0.6 g L-cysteine dissolved in pH 4.5 citric acid buffer. 9. Solution, 100 mL containing 2 g edaravone, 40 g HP-βCD, 1.2 g sodium bisulfite, and 0.6 g L-cysteine dissolved in pH 4.5 citric acid buffer. 10. Solution, 100 mL containing 2 g edaravone and 40 g HP-βCD dissolved in pH 4.5 citric acid buffer.
Wang et al., 2018 [9]; Wang et al., 2019 [59]	Sublingual edaravone tablets	11. Tablet (80 mg) with 30 mg edaravone, 41.2 mg lactose, 4 mg copovidone, 4 mg croscarmellose sodium, and 0.8 mg magnesium stearate. 12. Tablet (80 mg) with 30 mg edaravone, 41.2 mg mannitol, 4 mg copovidone, 4 mg croscarmellose sodium, and 0.8 mg magnesium stearate. 13. Tablet (70 mg) with 30 mg edaravone, 30.2 mg mannitol, 1.4 mg hydroxypropyl methylcellulose, 7 mg crospovidone, 0.7 mg silica, and 0.7 mg magnesium stearate. 14. Tablet (250 mg) with 30 mg edaravone, 186 mg mannitol, 10 mg hydroxypropyl methylcellulose, 20 mg crospovidone, 2 mg silica, and 2 mg magnesium stearate. 15. Tablet with 30 mg edaravone for human trial (composition unknown).
Li et al., 2024 [58]	Orally disintegrating tablet	16. Tablet with 60.00 mg edaravone, 1.60 mg Aeroperl^®^ 200, 125.9 mg mannitol, 1.0 mg citric acid, 0.50 mg sodium dodecyl sulfate, 4.00 mg croscarmellose sodium, and 8.00 mg stearic acid. 17. Tablet (60 mg) with 90.00 mg edaravone embedding substance, 1.00 mg sodium dodecyl sulfate, 171.0 mg mannitol, 11.0 mg crospovidone, and 8.00 mg sodium stearyl fumarate. Edaravone embedding substance is prepared by spraying carrier solution, containing 10.0 g cysteine hydrochloride, 200.0 g Eudragit^®^ E 100, and 700 g purified water, onto 400.0 g raw edaravone in multifunctional fluidised bed, adding 18.0 g Aeroperl^®^ 200, then drying.
	Orally dissolving film	18. Oral film (60 mg) containing 90.00 mg edaravone embedding substance, 4.00 mg colloidal silicon dioxide, 5.00 mg salcaprozate sodium, 68.00 mg polyvinyl alcohol, 34.00 mg hydroxypropyl methylcellulose, 1.00 mg polysorbate, and 3.00 mg sucralose, prepared by mixing components in purified water, then coating, drying, and cutting oral film. Edaravone embedding substance is as described in (16).
Peroral and oral mucosal formulations for acute ischaemic stroke patients		
Wang, 2021 [66]	Sublingual tablet with (+)-2-borneol as adjuvant	19. Tablet with 5 mg edaravone, 1 mg (+)-2-borneol, 2.4 mg mannitol, 3.7 mg lactose, 0.5 mg copovidone, 0.7 mg croscarmellose sodium, and 0.1 mg magnesium stearate. 20. Tablet with 5 mg edaravone, 1 mg (+)-2-borneol, 1 mg mannitol, 2.7 mg microcrystalline cellulose, 0.5 mg copovidone, 1.2 mg croscarmellose sodium, 0.2 mg silicon dioxide, and 0.1 mg magnesium stearate.
Li et al., 2018 [67]	Gastric retention pellets and enteric-coated pellets	21. Gastric retention pellets prepared by extrusion–spheronisation of mixture containing by weight 10% edaravone, 70% iron powder, 10% microcrystalline cellulose, 9% lactose, 1% sodium hydrogen sulfite, and 2% water. 22. Enteric-coated pellets prepared by extrusion–spheronisation of mixture containing 10% edaravone, 65% microcrystalline cellulose, 24% lactose, 1% sodium hydrogen sulfite, and 10% water, then coated by bed coating with Eudragit RS30D–Eudragit RL30D (5:1 *w*/*w*) until weight gain of 25%.

A quality target product profile (QTPP), summarised in Table 3, is proposed based on the analysis of Radicava^®^ and Radicava ORS^®^ as well as Rilutek^®^, Tiglutik^®^, and Exservan^®^. The QTPP aims for an edaravone product with a high oral BA, preferably over 90%, achieved through rapid in vivo dissolution and bypassing first-pass metabolism to ensure acceptable drug efficacy without increasing dosing frequency. Solid products are preferred over liquids, due to their superior storage stability. Additionally, tablets of appropriate size and dispersion rate that facilitate administration with minimal manipulation and cater to the different stages of MND pathophysiology, including when patients rely on feeding tubes, are desirable. The formulation design should support the ease of use and patient adherence to therapy and address the specific needs of the MND population. The manufacturing process should utilise low-cost production techniques to provide greater equity of access. This QTPP forms the basis of our review of the published alternative oral edaravone formulations, which are summarised in the following subsections.

### 6.2. Peroral Administration

#### 6.2.1. Inclusion Complexation with Water-Soluble Cyclodextrins

Ren et al. [60,61,62] at the Nanjing Normal University developed a series of water-soluble edaravone inclusion complexes for oral administration. Cyclodextrins are cyclic oligosaccharides able to accommodate appropriately sized drug molecules in their relatively hydrophobic cavity to improve drug solubility and dissolution in aqueous media [68]. Ren et al. prepared inclusion complexes of edaravone with unmodified β-cyclodextrin (βCD), hydroxypropyl-β-cyclodextrin (HP-βCD), sulfobutyl-β-cyclodextrin (SBE-βCD), and a novel βCD derivative, HP-SBE-βCD (Formulation 1, Table 2). The water-soluble βCD derivatives, HP-βCD and SBE-βCD, are preferred for solubilising drugs in liquid and solid medicinal products for oral and IV administration because they do not change the intrinsic biological properties of the drugs and are well tolerated in humans [69]. The novel HP-SBE-βCD was developed by the group for its high solubility, strong edaravone association, favourable toxicity profile, and ability to enhance the intestinal permeability of edaravone.

Phase-solubility studies suggest different degrees of edaravone association in the order of βCD < HP-βCD < SBE-βCD ~ HP-SBE-βCD (K_a_ (M^−1^, pH 6.86) = 639.1, 1331.3, 2346.6, and 2311.9, respectively). The edaravone–cyclodextrin association was pH-dependent and was more favourable at pH 6.86 than at pH 4.52. Further physicochemical studies performed at pH 6.86 showed linear increases in edaravone solubility with increasing concentrations of HP-βCD (y = 0.9552x + 0.013) and HP-SBE-βCD (y = 0.9678x + 0.0305). The inclusion complexes were fine powders containing a 1:1 molar ratio of edaravone–cyclodextrin, prepared by mixing an edaravone solution in ethanol with an aqueous cyclodextrin solution followed by freeze drying. Edaravone–HP-βCD (1 g edaravone to 8.85 g HP-βCD) powder containing 400 mg edaravone completely dissolved within 2 min in 100 mL of water at 37 °C at 100 rpm, whereas the equivalent free edaravone powder required a longer time for dissolution, and even then, only 50% had dissolved after 60 min. The edaravone–HP-SBE-βCD powder showed slower dissolution that was also pH-dependent, with 150 mg of the powder requiring ~20 min to dissolve in phosphate-buffered saline (PBS) at pH 7.4 while only about 60% had dissolved at pH 2 PBS after 6 h. The enhancement in edaravone solubility and dissolution in aqueous media was attributed to the improved wettability and conversion of crystalline edaravone to amorphous form when complexed with the cyclodextrins. 

The inclusion complexes were more stable than free edaravone. Stored as a powder in amber glass bottles, the edaravone–HP-βCD and edaravone–SBE-βCD inclusion complexes showed low degradation rates of no more than 2% after 10 days at 40 °C, compared to 5% for free edaravone. When dissolved in 0.9% sodium chloride solution and stored at room temperature for 24 h, the edaravone–HP-βCD complex formulation (concentration unknown) retained more than 96% residual drug content compared to less than 90% for free edaravone. 

Edaravone permeability across the jejunum epithelium of Sprague Dawley (SD) rats was improved when co-administered with HP-SBE-βCD or complexed with HP-SBE-βCD, and this was attributed to the HP-SBE-βCD-mediated inhibition of the P-glycoprotein efflux pump (Pgp). However, unlike specific Pgp inhibitors like verapamil, the inhibitory activity of HP-SBE-βCD was through a more generalised effect on the enterocyte membrane that included depletion of membrane cholesterol and modification of lipid raft structure.

To determine oral BA, the inclusion complexes were administered to SD rats by intragastric administration as aqueous solutions (concentration and vehicle unknown) to provide an edaravone dose of 27 mg/kg. Relative BA (F_rel_) determined against a suspension of edaravone (concentration unknown, vehicle: 0.5% carboxymethylcellulose sodium (CMC-Na), edaravone dose 27 mg/kg) was 1722.9% for edaravone–HP-βCD, 1324.8% for edaravone–SBE-βCD, and 1029% for edaravone–HP-SBE-βCD, as summarised in Table 4. Absolute BA (F_abs_) relative to a commercial IV edaravone solution (Bicun^®^, composition not given, dose 9 mg/kg) was 5.23% for the edaravone suspension, 71.6% for edaravone–HP-βCD, 55.0% for edaravone–SBE-βCD, and 53.8% for edaravone–HP-SBE-βCD (Table 4). 

In summary, Ren et al. have shown that edaravone forms inclusion complexes with HP-βCD, SBE-βCD, and HP-SBE-βCD that significantly improved its aqueous solubility and dissolution. There are, however, several issues that require attention and resolution. The resultant powders were recommended to be administered as tablets and capsules, which could prove challenging for MND patients to swallow. The inclusion complexes may be better administered as aqueous solutions, as was applied for the BA assessment; however, as the stability data reported only 24 h storage of the edaravone–HP-βCD inclusion complex in 0.9% sodium chloride, the long-term storage stability of the inclusion complexes in a liquid vehicle appropriate for oral administration in MND patients will need further investigation. Hiroshige et al. [70] demonstrated that complexation with βCD protected edaravone against oxidation in a βCD-concentration-dependent manner; however, in a pH 8 Universal buffer, edaravone (0.04 mM) in the presence of the highest βCD concentration (16 mM) was still completely degraded by 12 h. For Ren et al.’s formulations, the F_rel_ for the inclusion complex solutions were impressive, but this was measured against a comparator suspension formulation with very low F_abs_, less than 10-fold than that of Radicava ORS^®^ (5.23% vs. 57%). Furthermore, the F_abs_ for the SBE-βCD and HP-SBE-βCD inclusion complexes were on a par with, if not lower than, the F_abs_ for Radicava ORS^®^. HP-SBE-βCD is also a novel excipient that Rong et al. [61] found in their study to have potential to cause cell membrane damage, despite earlier investigations that indicated HP-SBE-βCD to have lower haemolytic and renal toxicity than βCD and HP-βCD [71]. Given its high molecular weight (>2200 g/mole), HP-SBE-βCD would be present at more than 12 times the mass of edaravone in the 1:1 molar ratio inclusion complex, raising concerns surrounding the safety of chronic use. Furthermore, the edaravone oral dose of 27 mg/kg applied for the BA study is equivalent to 1890 mg for a 70 kg human adult, which is significantly higher than the Radicava ORS^®^ dose of 105 mg and when translated to volumes of inclusion complex solutions required could potentially pose swallowing challenges for MND patients.

#### 6.2.2. In Vivo Nanosystems by Pre-Solubilising Edaravone in Lipids, Co-Solvents, and Surfactants

A research group in South Australia, led by Zhou and Garg [8,52,56,63], developed nano-formulations to enhance the oral BA of edaravone. They pre-dissolved edaravone in lipid, co-solvent, surfactants, and combinations thereof prior to oral administration. The group performed extensive pre-formulation studies to select excipients that improve edaravone BA, form nanosystems spontaneously in aqueous media, are safe, and are compatible with gelatine capsules. A wide range of formulations have been described, and only those that were further characterised (Formulations 2–5, Table 2) were reviewed. 

Lipid-based liquid SMEDDS (L-LNS), prepared by dissolving edaravone in a vehicle of 30% (*w*/*w*) of oil (Capryol^™^ PGMC), 50% of surfactants (1:0.8:0.2 Cremophor^®^ RH 40–Labrasol^®^–D-α-tocopheryl polyethylene glycol 1000 succinate (TPGS 1000)) and 20% of co-surfactant (Transcutol P^®^), was developed (Formulation 2, Table 2) [52,56]. The maximum edaravone loading was 6.45% *w*/*w*. The L-LNS spontaneously self-emulsified into oil-in-water (o/w) nanoemulsions in aqueous media, improved drug dissolution and stability in simulated GI fluids, and facilitated absorption that bypasses first-pass metabolism. The L-LNS self-emulsified in 32 s, forming 16.25 nm oil drops without phase separation or drug precipitation on dilution with simulated gastric (SGF) and intestinal (SIF) fluids. To ease handling, improve stability, and facilitate portability, the L-LNS was adsorbed onto Aerosil^®^ 200 particles and freeze-dried to form a powder (S-LNS) (Formulation 3, Table 2). The optimised S-LNS consisted of 1:1 *w*/*w* of L-LNS–Aerosil^®^ 200, with edaravone in amorphous form, and it maintained nano-size attributes when diluted. Compared to an edaravone suspension (0.5% CMC-Na as vehicle), L-LNS released edaravone faster in SGF (86.45% vs. 96.14% in 5 min, *p* < 0.05), while S-LNS required 30 min for complete drug release versus 15 min for the suspension and L-LNS. In SIF, the suspension had slower edaravone release than in SGF, likely due to the formation of soluble edaravone salts by hydrochloric acid in the SGF. Nevertheless, L-LNS and S-LNS had similar edaravone release profiles in both fluids. Oral BA in SD rats showed F_rel_ of 1079% for L-LNS and 929% S-LNS compared to a 30 mg/mL suspension, though F_abs_ was not reported for all formulations (Table 4). 

To reduce the high surfactant requirement of L-LNS, the group further developed self-nanomicellising solid dispersions (SNMSDs; Formulation 4, Table 2) [63]. The SNMSD was prepared by dissolving edaravone in an ethanolic solution of Soluplus^®^, evaporating the ethanol, and crushing the film into a powder. Soluplus^®^ was selected for its superior solubilisation capacity and its ability to form micelles at concentrations of 7.6 mg/L or higher in aqueous media. The optimised SNMSD was a solid solution comprising amorphous edaravone–Soluplus^®^ at 1:5 *w*/*w* and had enhanced edaravone solubility in both SGF and SIF. Storage at 40 °C/75% RH for 8 weeks did not change the drug content and aqueous dissolution profiles of the SNMSD. The SNMSD powder (equivalent to 15 mg edaravone) dissolved in 10 mL of water to yield dispersions of Soluplus^®^ micelles (73.46 nm) that entrapped ~96% of the edaravone, with a final drug load of 0.143% *w*/*w*. The micelles protected edaravone from glucuronidation (2.4-fold decrease) and increased intestinal permeability by 2.73-fold compared to free edaravone in the rat. An oral BA study in SD rats showed that the SNMSD had F_rel_ of 1024% at 46 µmol/kg, 1608% at 138 µmol/kg, and 1478% at 414 µmol/kg against the suspension comparator. F_abs_ was again not reported for all formulations (Table 4).

This group also developed a simple aqueous solution of edaravone (30 mg/mL) in Labrasol^®^ and 0.05 M citric acid buffer (pH 4) in an unspecified optimised ratio (Formulation 5, Table 2) [8]. The acidic buffer was chosen because edaravone was more stable at pH 2–4 than at a higher pH. Labrasol^®^ is a water-miscible surfactant that could increase cell membrane lipid fluidity at concentrations of 20% or higher [72]. Labrasol^®^ forms micelles at 0.01% *w/v*, with the diameter reaching 20 nm at 25% *w/v*, and it enhanced edaravone solubility in 100% Labrasol^®^ to 62.48 mg/mL. Labrasol^®^ at 1% inhibited glucuronidation of edaravone by 88.78% and increased its intestinal permeability by 3.73-fold compared to free edaravone. No significant changes in drug content or apparent precipitation were observed when the formulation was stored under nitrogen at 40 °C/75% RH for 1 month. F_rel_ determined in SD rats against the edaravone suspension in 0.5% CMC-Na was 575% for the Labrasol^®^–edaravone solution at the edaravone dose of 30 mg/kg (Table 4). This was attributed to edaravone being completely solubilised in the Labrasol^®^ formulation, while the suspension in 1.5 mL of vehicle contained only 1.89 mg/mL of solubilised drug. F_abs_ was not reported.

Overall, the South Australia group has developed 11 formulations for oral edaravone delivery, including powders, liquids, and semi-solids, which improve the solubility and stability of edaravone. These formulations are recommended for gelatine capsule or liquid delivery. The nano-formulations had superior stability to free edaravone. There are, however, some gaps in the data provided. The edaravone strength, quantity of formulations required to deliver the edaravone doses, and the vehicle for delivering the formulations in the BA studies are not always published. Given the high solubiliser–edaravone ratios in these formulations, relatively large quantities of formulations could be required to provide the recommended edaravone dose, raising concerns regarding swallowability and excipient safety. Indeed, the authors themselves acknowledged the risks associated with the high surfactant concentrations in the SMEDDS formulations [63]. While the F_rel_ data obtained for the nanosystems are promising, the lack of F_abs_ data necessitates caution, partly because the edaravone doses and control suspension formulation were comparable to those of Ren et al. There remain unmet patient-centred considerations for the nano-formulations, including descriptions of the practical dose, dosing regimen, and administration method for MND patients. Moreover, as surfactants and oils are not known to be pleasant tasting, palatability assessment should be considered for those nano-formulations intended to be administered without encasement in capsules. 

#### 6.2.3. Powder (TW001) for Preparing Alkaline Oral Solutions of Edaravone

Van der Geest and Moolenaar [55,65] patented an edaravone solution formulation based on findings that the IV Radicut^®^ solution (30 mg edaravone/20 mL) exhibited higher than expected F_abs_ of 34% when administered orally to beagle dogs. IV Radicut^®^ is available in two volumes: 100 mL of solution containing 30 mg edaravone ready for infusion (Radicut^®^ Bag for IV infusion 30 mg) and 20 mL of solution containing 30 mg edaravone to be diluted with physiological saline before infusion (Radicut^®^ injection 30 mg) [46,73]. For both products, like Radicava^®^, each unit also contains sodium bisulfite 20 mg, L-cysteine hydrochloride 10 mg, and sodium hydroxide/phosphoric acid to adjust pH to 3–4.5 [74]. Van der Geest and Moolenaar developed a powder formulation (TW001) that consisted by weight of 9.3% micronised edaravone, 56.8% mannitol, 33.3% sodium orthophosphate, and 0.5% sodium lauryl sulfate (Formulation 7, Table 2). Mannitol is a filler [75], while sodium lauryl sulfate is a surfactant solubiliser [76]. Surprisingly, the pair prioritised solubility over stability; although it is well known that edaravone has higher propensity to degrade at alkaline pH [8], the pair chose to use an alkalising agent (sodium orthophosphate) to provide a final pH of 6.8–8.5 to enhance the aqueous solubility and dissolution. The stability issue was however addressed by formulating TW001 as a powder to be rendered into a solution no more than 3 h prior to oral administration. The pair did also propose kits of 14 individually sealed units each containing 25–50 mL of solution equivalent to 40–120 mg edaravone; however, the design and characterisation of this liquid formulation have not been further elaborated. 

The PK study was first performed in four beagle dogs using a formulation that contained a lower edaravone–mannitol ratio (8.0:58.2%) (Formulation 6, Table 2). The dogs received 750 mg of the formulation (60 mg edaravone) administered orally in 50 mL water and an IV infusion of two Radicut^®^ ampoules (60 mg edaravone) [64]. The F_abs_ was 31%. A higher F_abs_ of 79% was obtained for TW001 in a single dose, randomised, two-period crossover Phase I trial involving 18 human participants who received 100 mL of oral solution containing 1.5 g of TW001 (140 mg of edaravone) and IV Radicut^®^ (60 mg edaravone). A second similar study was conducted which also monitored edaravone metabolites, and the F_abs_ was 93%, with no differences in sulfate and glucuronide conjugate-to-parent drug observed between the IV and oral formulations (Table 4) [55,65]. 

The TW001 formulation is simple in formulation design and preparation method, and, unlike Radicava ORS^®^, it has a simpler treatment regimen of once-daily dosing without interruptions. The recommendation of drug-free periods for Radicava^®^ administration might have stemmed from reports of serious side effects (acute kidney failure and liver dysfunction) following edaravone use in acute ischemic stroke. However, no such side effects have been observed in all placebo-controlled studies for edaravone as an ALS/MND treatment. Additionally, while the drug-free periods may reduce the burdens of daily IV infusion for Radicava^®^, it is not relevant for oral edaravone formulations that can be self-administered in a home setting. Having a complex dosing regimen could in fact reduce therapeutic compliance, with the 2-week drug-free periods also having the potential to cause ROS accumulation with adverse effects on motor neurones [7]. TW001 is formulated to be administered as a liquid, which facilitates swallowing; however, the edaravone dose is contained in a volume of 100 mL, which could be challenging to consume for dysphagic MND patients. Furthermore, as edaravone is unstable in an alkaline pH [8], patients will have to prepare fresh solutions of TW001 for every edaravone dose, which is inconvenient, prone to dosing errors, and difficult to achieve for patients with limited mobility. Nonetheless, of all the published novel oral edaravone formulations, TW001 is the most advanced in human testing. Unfortunately, despite its great promise, disappointing topline results were announced in January 2024 for the multicentre, double-blind, randomised, placebo-controlled Phase III ADORE study, in which ALS patients were dosed with TW001 (100 mg edaravone) once daily for 48 to 72 weeks [77]. Although it was shown to be safe and well tolerated, the formulation did not show significant benefit compared to placebo, and the extended trial was discontinued as a result.

### 6.3. Oral Mucosal Administration

#### 6.3.1. Solutions with Cyclodextrins and Antioxidants

In a bid to bypass first-pass metabolism altogether, Sato et al. [57] investigated the delivery of edaravone via the oral mucosa. Solutions in pH 4.5 citric acid buffer were prepared to contain edaravone and HP-βCD in the weight ratio of 1:20, with and without L-cysteine and sodium bisulfite (Formulations 8–10, Table 2). Solution 8 (with 1% *w/v* edaravone) was formulated to provide a 0.5 mg edaravone/rat dose, while solutions 9 and 10 (both had 2% *w/v* edaravone) provided a 1 mg edaravone/rat dose. Solutions 8 and 9 contained L-cysteine (0.6% *w/v*) and sodium bisulfite (1.2% *w/v*), whereas solution 10 did not contain the antioxidants. All solutions were administered by spraying into the oral cavity of male Wistar rats (0.05 mL per rat). Edaravone absorption for solutions 8 and 9 was immediate (T_max_ 3–5 min) and produced a high C_max_ that was dose-dependent. The F_abs_ for both solutions determined against solution 8 administered by IV were 100% (Table 4). In contrast, solution 10, which lacked L-cysteine and sodium bisulfite, had a delayed T_max_ (10 min) and lower C_max_ (F_abs_ not provided). Sato et al. suggested that L-cysteine could enhance transcellular membrane transport by interacting with the keratin of basal cells in the oral mucosal, while both L-cysteine and sodium bisulfite could inhibit the systemic metabolism of edaravone by stabilising its ketone-reactive site to prevent keto–enol transformation. 

This study is interesting in two aspects. Firstly, it demonstrated the feasibility of delivering edaravone as an oral mucosal formulation with F_abs_ of 100%. Secondly, it demonstrated that L-cysteine and sodium bisulfite could play critical roles in moderating the pharmacokinetics of edaravone formulations. L-cysteine and sodium bisulfite are present also in Radicava^®^ and Radicava ORS^®^, and they might well be the reason for why the F_abs_ for Radicava ORS^®^ is 57% while the F_abs_ for the oral suspension comparator prepared by Rong et al. [61], which did not contain L-cysteine and sodium bisulfite, was only 5.23%. Sato’s solutions 8 and 9 are promising formulations for dysphagic patients as they are administered as fine sprays into the oral cavity, provided an appropriate spray device is available. The solutions should also be subject to taste assessment and long-term storage stability analysis at ambient conditions.

#### 6.3.2. Sublingual Tablets

Wang et al. [9,59] at Nanjing Lifecare Pharmaceutical Co. Ltd. aimed to develop sublingual edaravone tablets that exhibited an acceptable stability, drug release rate, and oral BA (Formulations 11–15, Table 2). The sublingual mucosa is a relatively thin epithelium located underneath the tongue on the floor of the mouth with abundant blood supply that bypasses first-pass drug metabolism in the liver and offers fast drug absorption [78]. Fourteen tablet formulations, all of which contained 30 mg of edaravone, were prepared by mixing to uniformity the powder ingredients, weighed according to specified formula, and compressing the final powder mix in a tablet press. The choice of excipients was critical, with the combination of mannitol as filler, hydroxypropyl methylcellulose as binder, and crospovidone as disintegrant (Formulations 13 and 14, Table 2) found to provide an optimal stability and drug release profile for the sublingual tablet.

The oral BA was determined for four 30 mg tablet formulations (Formulations 11–14, Table 2) by inserting a tablet under the tongue of a male beagle dog and fixing the mouth of the dog for 12 min to prevent swallowing of the tablet or excess saliva. The F_abs_ for tablet Formulations 11 and 12 determined against IV edaravone (10 mg/5 mL solution; edaravone dose 24 mg) were 64% and 73%, respectively. The lower-than-expected BA was attributed to incomplete drug release from the tablets. In contrast, the F_abs_ for tablet Formulations 13 and 14 were 126% and 100%, respectively (Table 4), leading the authors to suggest that these tablets could replace IV edaravone in a clinical setting. A high F_abs_ of 92% was also obtained in a randomised two-way crossover trial performed in 10 healthy male volunteers who received, at least 24 h apart, 30 mg of IV edaravone and a 30 mg sublingual edaravone tablet (Formulation 15, Table 2). The tablet composition for the human trial was not revealed except that it contained 30 mg edaravone, had excipients of “low moisture and low oxygen content”, the synthesis/manufacturing processes avoided water, heat, and light, the packaging was a polyester/aluminium/polyethylene-laminated film to minimise oxygen and light exposure, and it was reportedly stable after 3 months of storage at 25 °C/60% RH. The F_abs_ for the tablet in beagle dogs was ~80% against IV infusion of 30 mg edaravone delivered over 15 min. In the human trial, the time to tablet dissolution was determined by examining the sublingual area of participants at specific time points post-administration. The tablet dissolution time was found to be at least 10 min for 70% of the participants, with one participant requiring 30 min for tablet dissolution. The tablet was however well tolerated. 

In summary, the sublingual tablet developed by Wang et al. for the human trial has highly promising F_abs_ compared to Radicava ORS^®^. Being a solid formulation, it offers dosing accuracy and convenient self-administration and had apparent stability at ambient storage conditions. Other advantages are that the inactive ingredients are common tablet excipients, and the manufacturing method can be readily scaled up using standard tabletting processes. It is exciting that the common tablet excipients in the tablet were effective in solubilising a 30 mg edaravone load in the small volume of liquid found in the oral cavity. Further investigation into the mechanism of action would be helpful to exploit this finding, given that these excipients are not known to solubilise water-insoluble drugs by simple mixing alone.

A challenge with sublingual drug delivery is the rapid production of saliva in the sublingual area, as this stimulates swallowing that leads to the drug being delivered to the lower GIT and negating the advantage of bypassing first-pass metabolism [78]. Sublingual tablets are therefore formulated to be small and have bland taste to reduce saliva production and to dissolve very quickly, before the tablet becomes dislodged or saliva is swallowed. For the oral BA study, Wang et al. had taken steps to prevent saliva swallowing; however, given the long dissolution time of the tablet in the oral cavity and the method employed, these steps would not be practical to implement in MND patients. Thus, the long dissolution time of the sublingual tablet would have to be addressed to ease sublingual administration in MND patients. An alternative is for the sublingual tablet to be applied for buccal delivery, as the buccal route may better tolerate tablets with a longer dissolution time than the sublingual route.

#### 6.3.3. Orally Disintegrating Tablets and Orally Dissolving Films

Li et al. [58] formulated an orally disintegrating tablet (ODT) and an orally dissolving film (ODF) of edaravone (Formulations 17–18, Table 2) by first developing a spray-dried powder. Edaravone (400 g) in a carrier solution comprising 10 g cysteine hydrochloride, 200 g Eudragit^®^ E100, and 700 g purified water was spray-dried with 18 g Aeroperl^®^ 200 as anti-adherent. The embedment of edaravone in the carrier rendered it amorphous to increase aqueous solubility, and it has the potential to reduce the bitter taste of the drug. The spray-dried powder was stable after 6 months storage at 40 °C and 75% RH. To prepare the ODT, 45 g of spray-dried powder (30 g edaravone) was pressed into an 8 mm tablet (each 60 mg) with 0.5 g sodium dodecyl sulfate, 85.5 g mannitol, 5.5 g crospovidone, and 4 g of sodium stearyl fumarate (Formulation 17, Table 2). The ODF was prepared by mixing 45 g of spray-dried powder with 2 g of Aeroperl^®^ 200, 2.5 g salcaprozate sodium, 34 g polyvinyl alcohol, 17 g hydroxypropyl methylcellulose, 0.5 g polysorbate, 1.5 g sucralose, and 152.88 g purified water, followed by drying and cutting into 60 mg squares measuring no more than 35 mm in length (Formulation 18, Table 2). Another ODT formulation was prepared by firstly freeze drying a 1000 mL aqueous solution of 30 g edaravone, 62.95 g mannitol, 0.5 g citric acid, 0.25 g sodium dodecyl sulfate, and 2 g croscarmellose sodium and pressing the freeze-dried powder with 0.8 g Aeroperl^®^ 200 and 4 g stearic acid into 8 mm tablets (Formulation 16, Table 2). 

The formulations were assessed against a commercial edaravone oral suspension. Based on the level of impurities detected, Formulation 16 was stable after 6 months of storage at 40 °C and 75% RH but was less stable than the oral suspension. Stability data were not provided for the ODT Formulation 17 and ODF formulation that contained the novel edaravone-embedded powder. All three formulations exhibited near-complete edaravone dissolution (≥99%) in 900 mL of acetate buffer (pH 4.0, 75 rpm) at 60 min, with the dissolution rate in the order of ODF > ODT Formulation 17 > ODT Formulation 16. The suspension comparator had the slowest rate of dissolution (95% at 60 min). The fast dissolution rate of ODF (84% at 5 min, 100% at 20 min) was attributed to both the thin layer of this film formulation and the embedment of edaravone in the carrier. The oral BA was determined in Japanese white rabbits (2.06–2.19 kg) using the following interventions: IV edaravone injection (1.5 mg/kg), oral edaravone suspension (2 mg/kg), and the novel formulations (2 mg/kg). The F_abs_ for Formulations 16–18 ranged from 64 to 74%, and all showed improved BA (>4-fold) compared to the suspension (Table 4). 

Compared to other formulators, Li et al. have considered a wider range of factors when designing the ODTs and ODF, including better storage stability, dosing accuracy, convenience, and ease of self-administration, as well as taste. The tablet manufacturing process for the ODTs is also readily amendable to scale up, although the pre-preparation of spray-dried edaravone-embedded powder might make it a less attractive method than that for the sublingual tablet of Wang et al. The F_rel_ for the ODTs and ODF were also less impressive than for the sublingual tablets, but this is to be expected, for unlike the sublingual tablets, the ODTs and ODF were designed to be swallowed and therefore would be exposed to first-pass drug loss in the GIT. However, ODT and ODF formulations are required to have rapid disintegration/dissolution in the mouth [79], which would be beneficial for MND patients, whereas such requirements are not specifically stated for sublingual and buccal tablets [80]. As Li et al. have not reported on the disintegration and dissolution times for their formulations in the mouth, it is difficult to state with certainty that they are an improvement upon the dissolution times for the sublingual tablets formulated by Wang et al. [9,59].

## 7. Oral Formulations of Edaravone Formulated for Patients with Ischemic Stroke

Edaravone was first approved for the treatment of acute ischaemic stroke [81], and therefore, there are also ongoing efforts to develop oral formulations of edaravone for patients in this disease group. Two formulations are highlighted here, one for peroral administration and another for sublingual administration.

Yantai Yenepharma developed sublingual tablets of edaravone (Formulations 19 and 20, Table 2) that was evaluated in acute ischaemic stroke patients [66,82]. The sublingual tablet formulations, interestingly, had a much lower oral BA compared to the sublingual tablet of Wang et al. As described in Table 2, the Yantai tablets contained a small 5 mg edaravone load, together with 1 mg (+)-2-borneol as a permeation enhancer, and typical tablet excipients, though the Fu et al. [82] article administered tablets of 30 mg edaravone and 6 mg (+)-2-borneol to acute ischaemic stroke patients. To perform the BA study in SD rats, the tablets were placed under the tongue of the rat, and the mouth of the animal was fixed for 30 min to prevent the tablet from falling off or sliding into the GIT. The sublingual tablet Formulations 19 and 20 (Table 2), despite having (+)-2-borneol as a permeation enhancer and showing complete drug release within a short time of 5 min, had F_abs_ of only 62.6% and 79.9%, respectively, when measured against an IV edaravone solution (each 5 mL contained edaravone 10 mg and (+)-2-borneol 2.5 mg, dosed at 8 mL/kg) (Table 4). It is not known if the difference in F_abs_ between these tablets and the sublingual tablet of Wang et al. was caused by the variations in formulation or the animal model and treatment protocol employed in the BA study. 

Li et al. [67] adopted a different approach by developing densified pellets that would be retained in the stomach long enough for edaravone to be released and absorbed in the stomach for patients with ischaemic stroke (Formulation 21, Table 2). The reasoning was that absorption in the stomach could reduce the Pgp efflux of edaravone, while the acidic pH could maintain the drug in nonionised form to enhance stability and facilitate stomach absorption. The gastric retentive pellets (700–1000 µm in size, 2.4 g/cm^3^) were prepared by the extrusion–spheronisation of a mixture comprising edaravone (10% *w*/*w*), dense iron powder (70%), microcrystalline cellulose (10%), lactose (9%), sodium bisulfite (1%), and water (~2 g for 100 g powder). X-ray imaging showed the pellets to settle in the stomach of SD rats, with no significant change in position for up to 3 h, and all pellets were discharged into the intestine in ~7 h. BA studies performed with an edaravone dose of 16 mg/kg in SD rats showed the F_abs_ to be 68.96% for the gastric retentive pellets, which is significantly higher than for edaravone solution (1.5 mg/mL in normal saline, F_abs_ 33.85%, 2.13 mL/200 g) and enteric-coated pellets (F_abs_ 7.64%; Formulation 22, Table 2) (Table 4). The commercial IV Bicun^®^ injection administered at 16 mg/kg was the comparator. Similar F_rel_ data against the solution formulation were obtained in the beagle dog model. The enteric-coated pellet (1.2 g/cm^3^) was prepared in a similar manner by extrusion–spheronisation using a mixture devoid of iron powder (edaravone 10%, microcrystalline cellulose 65%, lactose 24%, sodium bisulfite 1%, and water ~10 g per 100 g powder). The resultant pellets were coated with EudragitRS30D–EudragitRL30D (5:1 *w*/*w*) to a coat weight of 25% *w*/*w*. The low F_abs_ for the enteric-coated tablet led the authors to suggest that absorption in the stomach may be superior to absorption in the intestine to boost the oral BA of edaravone. 

## 8. Discussion

This review has highlighted several interesting and promising attempts at developing oral edaravone medicinal products for MND patients. Table 2 and Table 4, respectively, describe the formulations’ composition and salient pharmacokinetic data, while Table 5 summarises the benefits and limitations of each approach. It is apparent from Table 5 that the formulations lack taste assessment and MND-patient-centred data. An ideal dosage form for edaravone is one that is patient-centred, offers convenient and accurate dosing, and can be self-administered in a home setting. The formulation would be stable over its shelf life, while also exhibiting high BA and low toxicity. Such a formulation is best met with a solid form, like a tablet, which would also provide a more stable platform for the labile edaravone molecule than a liquid vehicle. The tablet should be of appropriate size that is easy to handle for patients with limited dexterity but not too big as to pose choking risks. A tablet formulation is preferred over a capsule as technologies are available to design a tablet formulation that will rapidly disintegrate, or better still, dissolve in the mouth. This is critical to facilitate administration in MND patients with increasing degrees of dysphagia as the disease progresses. 

It would be preferable for the tablet to be designed for sublingual administration to bypass first-pass metabolism, Pgp efflux, and the extremes of pH encountered in the lower GIT. The oral cavity, however, presents the challenge of having only a low volume of saliva available to dissolve the edaravone dose [83]. It will therefore be critical for the formulation design to incorporate safe strategy(ies) to ensure that the edaravone dose is not only completely solubilised in the oral cavity but that solubilisation is achievable within a short time. A tablet that dissolves in the oral cavity must also have excipients of acceptable taste to increase medication compliance. The tablet formulation of Wang et al. offers a template design for sublingual delivery, although further optimisation may be required to reduce its dissolution time in the oral cavity. Taste assessment should also be performed.

Alternatively, buccal administration, which is more tolerant of tablets with a long dissolution time, may be explored for edaravone delivery. The buccal mucosa is located on the inside lining of the cheeks and lips. Much like the sublingual mucosa, the buccal mucosa comprises non-keratinised epithelium which is more permeable than other types of oral mucosa. It is also located close to the parotid salivary glands that are responsible for the saliva flow into the buccal areas. The sublingual mucosa is comparatively thinner and more vascularised than the buccal mucosa, making it more permeable and suited for rapid drug absorption. However, frequent tongue movements and high salivation in the sublingual region reduce the contact time between the mucosa and drug product [78]. Therefore, sublingual tablets must disintegrate rapidly and preferably contain small drug doses (20 mg or less) to ensure complete drug absorption via the sublingual mucosa [84]. On the other hand, the buccal mucosa, while less permeable, is relatively immobile, making it suitable for prolonged drug absorption [85]. The buccal area also experiences high salivation that may promote swallowing, but this can be overcome with mucoadhesive delivery systems [86]. Edaravone may be incorporated with mucoadhesive excipients to produce a formulation that is able to adhere to mucosal membranes to increase the contact time between the drug and mucosal surface [87]. 

Lastly, none of the reviewed formulations showed evidence of having been designed, fabricated, and assessed with consumer engagement and participation. Due to the unique challenges of MND patients, it would be critical to determine the medication preferences of people with lived experience of MND, whether patients or carers, to ensure the final oral formulation is fit for purpose. Further research in this area is warranted, especially if a dosage form for buccal administration, which is relatively uncommon compared to peroral administration, is to be formulated for edaravone to treat MND patients. 

## 9. Conclusions

In conclusion, 14 texts published by six research groups on 18 novel oral formulations of edaravone for the treatment of MND have been reviewed. All the formulations were able to improve the aqueous solubility and oral BA of edaravone compared to an edaravone suspension. All formulations were assessed to be stable in storage at ambient temperature; however, the period of storage stability testing was variable, from short periods of 24 h to 3 months, while TW001, ODT Formulation 17, and ODF Formulation 18 had no published stability data. A common limitation of the published formulations is the lack of MND-patient-centred data. Except for TW001, no other formulations have been trialled in MND patients. To meet the QTPP of an oral edaravone formulation for MND patients, it is recommended that a tablet of appropriate size and with acceptable taste and stability be designed for the effective sublingual or buccal absorption of edaravone. This tablet should be designed with input from the MND community.

## Figures and Tables

**Table 3:** Quality target product profile (QTPP) for edaravone formulations to treat motor neurone disease (MND).

Attribute	Targets	Comments
Target population	Adults diagnosed with MND	Focus on adult patients due to the nature of MND.
Route of administration	Peroral and oral mucosal (e.g., sublingual)	Oral route has wide acceptance and allows for administration in a home setting without involvement of healthcare professionals, offering convenience to both patients and caregivers; sublingual tablets are small and rapidly dissolving, which benefit patients with swallowing difficulties; sublingual epithelial offers rapid absorption free from first-pass metabolism.
Target PK profile	Immediate release, high BA	Immediate release to enable rapid and complete dissolution of drug dose; BA higher than 90% by design to counter first-pass metabolism.
Dosage form	Small tablets that rapidly disintegrate in the mouth	Solid dosage form is more stable than liquid formulations, allowing for longer shelf life and reducing frequency of prescription refills. Small tablets that rapidly disintegrate or dissolve within 3 min to facilitate oral administration in patients with difficulty swallowing.
Dose	Based on clinical efficacy and safety data	Dosing regimen designed to maintain efficacy of edaravone without necessitating more frequent administration than the current regimen. The goal is to optimise patient adherence and minimise treatment burden.
Excipients and manufacturing	GRAS excipients, cost-effective production	Selected excipients are established pharmaceutical ingredients with no specific safety concerns for the MND population; manufacturing process to utilise low-cost techniques and materials.
Patient acceptability	High acceptability	Tablets designed to avoid causing difficulty in swallowing to enhance acceptance by patients in the early and mid-stages of MND. Formulation prioritises ease of administration, incorporating patient feedback to accommodate the varying abilities and preferences within MND patient group, thereby enhancing overall treatment adherence. Formulation does not have unpalatable taste.
Administration considerations	Ease of administration, minimal preparation	Tablets designed to minimise the need for manipulation for administration, particularly for end-stage MND patients relying on PEG tubes. This requires tablets to be easily dissolved in the liquid for PEG feeding without compromising drug stability or efficacy.

PK: pharmacokinetic; BA: bioavailability; GRAS: generally recognised as safe; PEG: percutaneous endoscopic gastrostomy.

**Table 4:** Absolute (F_abs_) and relative (F_rel_) oral BA and PK activity of the alternative edaravone formulations in different species.

Formulation Type	In Vivo Model	F_abs_	F_rel_	PK Notes	Ref.
Peroral formulations for MND patients					
Edaravone complexed with βCD	Rat (*n* = 30)	53.8–71.6%	1029–1722% relative to suspension ^1^	Oral administration extended half-life (t_1/2_) to as long as 3.48 ± 0.31 h, surpassing the IV t_1/2_ of 0.20 ± 0.15 h.	[60,61]
Liquid SMEDDS	Rat (*n* = 12)	-	1080% relative to suspension ^1^	Liquid SMEDDS increased t_1/2_ to 78.49 ± 2.53 min, compared to 58.31 ± 3.52 min for suspension ^1^.	[52,56]
Solid SMEDDS	Rat (*n* = 12)	-	930% relative to suspension ^1^	Solid SMEDDS showed a prolonged t_1/2_ of 85.12 ± 3.81 min vs. suspension ^1^ t_1/2_ of 58.31 ± 3.52 min.	[52,56]
Solid dispersion	Rat (*n* = 24)	-	1024–1608% relative to suspension ^1^	Solid dispersion form exhibited extended t_1/2_ of 73.26 ± 6.81 min, exceeding suspension ^1^ t_1/2_ of 58.31 ± 3.52 min.	[56,63]
Aqueous solution with co-solvent/solubiliser	Rat (*n* = 12)	-	575% relative to suspension ^1^	Co-solvent-based liquid formulation lengthened t_1/2_ to 75.98 ± 6.53 min versus 58.31 ± 3.52 min for suspension ^1^.	[8,56]
Alkalinised solution	Dog (*n* = 4)	30.6%	-	-	[64]
	Human (*n* = 18)	79–93%	-		[55,65]
Oral mucosal formulations for MND patients					
Aqueous solution with HP-βCD and antioxidants	Rat (*n* = 9)	100%	-	Orally administered formulation extended α t_1/2_ to 12.8 ± 2.2 h from 3.0 ± 0.7 h for IV. However, β t_1/2_ did not differ significantly.	[57]
Sublingual edaravone tablets	Dog (*n* = 14)	64–100%	-	-	[59]
	Human (*n* = 10)	92%	-	Oral form in humans showed a shorter t_1/2_ of 2.83 ± 0.73 h compared to IV t_1/2_ of 3.04 ± 0.62 h.	[9]
Orally disintegrating tablets	Rabbit	64–71%	401–440% relative to suspension ^2^	-	[58]
Orally dissolving film	Rabbit	74%	460% relative to suspension ^2^	-	[58]
Peroral and oral mucosal formulations for acute ischaemic stroke patients					
Sublingual tablets with (+)-2-borneol as adjuvant	Rat (*n* = 16)	62.6–79.9% (plasma) 28.5–30.1% (brain tissue)	-	-	[66]
Gastric retention pellets	Rat (*n* = 18)	68.96 ± 5.66%	203.70% relative to solution ^3^	Pellets displayed a t_1/2_ of 3.54 ± 0.37 h in rats, outlasting IV t_1/2_ of 2.94 ± 0.30 h and similar to solution ^3^ with a t_1/2_ of 3.48 ± 0.79 h.	[67]
	Dog (*n* = 6)	-	211.02 ± 9.29% relative to solution ^3^	In beagles, t_1/2_ is 4.59 ± 0.22 h versus 4.13 ± 0.36 h for solution ^2^.	[67]
Enteric-coated pellets	Rat (*n* = 18)	7.64 ± 1.03%	22.58% relative to solution ^3^	Pellets displayed a t_1/2_ of 3.40 ± 0.32 h in rats.	[67]

^1^ Edaravone suspension in 0.5% carboxymethylcellulose sodium (CMC-Na). ^2^ Edaravone oral suspension (Mitsubishi Tanabe Pharmaceutical Co., Ltd. (Osaka, Japan); 105 mg/5 mL) exhibited absolute BA of 16% in the same study in rabbits. ^3^ Edaravone solution in normal saline.

**Table 5:** Summary of the benefits and limitations of the published alternative oral edaravone formulations for MND patients.

Formulation Type	Benefits	Limitations	Ref.
Peroral formulations for MND patients			
Edaravone complexed with βCD	Rapid (<2 min) aqueous dissolution. Low (<2%) degradation after 10 days storage at 40 °C. Stable (96% drug content) in aqueous solution after 24 h at room temperature. Improved edaravone permeability across jejunum epithelium. Very high (up to 1722%) F_rel_ vs. suspension ^1^.	Long-term stability in liquid not assessed. F_rel_ measured vs. suspension ^1^ with very low F_abs_ (5.23%). Little/no improvement in F_abs_ vs. Radicava ORS^®^. High βCD amounts: safety concerns over chronic use. Impractically high edaravone doses used in BA study. Administration poses swallowing challenges.	[60,61,62]
Liquid SMEDDS	Rapid (~5 min) dissolution in simulated gastric (SGF) and intestinal (SIF) fluid. High (1079%) F_rel_ vs. suspension ^1^. Considered safe in cell viability study.	No F_abs_ reported. F_rel_ measured vs. suspension ^1^. High surfactant amount: gastrointestinal (GI) irritation with chronic use. Limited practical dosing information provided. Administration poses swallowing challenges. Taste concerns regarding surfactants/oils.	[52,56]
Solid SMEDDS	High (929%) F_rel_ vs. suspension ^1^. Solid: better stability, easier handling, and more portable than liquids. Considered safe in cell viability study.	Slow (30 min) dissolution in SGF and SIF. No F_abs_ reported. F_rel_ measured vs. suspension ^1^. High surfactant amount: GI irritation with chronic use. Limited practical dosing information provided. Administration poses swallowing challenges. Taste concerns regarding surfactants/oils.	[52,56]
Solid dispersion	Stable for 8 weeks at 40 °C/75% relative humidity (RH). Stable after 20-fold dilution and 24 h storage in SIF and SGF at 40 °C/75% RH. Improved (17.53-fold) edaravone aqueous solubility vs. crude edaravone. Enhanced dissolution in SGF and SIF vs. crude edaravone. Decreased (2.4-fold) edaravone glucuronidation. Increased (2.73-fold) edaravone intestinal permeability. High (up to 1608%) F_rel_ vs. suspension ^1^. Considered safe in cell viability study.	No F_abs_ reported. F_rel_ measured vs. suspension ^1^. Limited practical dosing information provided. Administration poses swallowing challenges. Taste concerns regarding surfactants/oils.	[56,63]
Aqueous solution with co-solvent/solubiliser	Inhibits edaravone glucuronidation. Enhanced edaravone aqueous solubility. Improved (3.73-fold) edaravone intestinal permeability. Stable for 1 month at 40 °C/75% RH. High (575%) F_rel_ vs. suspension ^1^. Safe in cell viability study.	No F_abs_ reported. F_rel_ measured vs. suspension ^1^. Limited practical dosing information provided. Administration poses swallowing challenges. Taste concerns regarding surfactants/oils.	[8,56]
Alkalinised solution	Enhanced edaravone aqueous solubility. High (up to 93%) F_abs_. No change in metabolism vs. IV. Solid form available: better stability, easier handling, and more portable than liquid. Simpler dosing regimen.	No stability analysis. Administration poses swallowing challenges. Inconvenient reconstitution prior to administration. Phase III study results: no efficacy as ALS treatment.	[55,64,65]
Oral mucosal formulations for MND patients			
Aqueous solution with HP-βCD and antioxidants	Very high (100%) F_abs_. Oral mucosal absorption: bypasses first-pass metabolism and potentially easier administration.	No stability analysis. Limited practical dosing information provided.	[57]
Sublingual edaravone tablets	Complete in vitro dissolution within 10 min. Stable for 3 months at 25 °C/60% RH. Very high (up to 100%) F_abs_. Sublingual absorption: bypasses first-pass metabolism and potentially easier administration. Solid tablet: dosing accuracy, better stability, easier handling, and more portable than liquids.	Long dissolution time (average of 10 min) in mouth: impractical for MND patient administration.	[9,59]
Orally disintegrating tablets	Formulation 16 is stable for 6 months at 40 °C and 75% RH. 92% dissolution within 10 min. Fast disintegration and easier administration. Solid tablet: dosing accuracy, better stability, handling, and portability than liquids.	Lower BA than oral mucosal formulations (F_abs_ = 64–71%). No storage stability data for Formulation 17. Disintegration time in mouth unknown.	[58]
Oral film	96% dissolution within 10 min. Fast dissolution, potentially easier administration. Solid film: dosing accuracy, potentially better stability and portability than liquids.	Lower BA than oral mucosal formulations (F_abs_ = 74%). No storage stability data. Dissolution time in mouth unknown. Ease of handling of thin films unknown.	[58]

^1^ Edaravone suspension in 0.5% carboxymethylcellulose sodium (CMC-Na).
